# 154. Antibiotic Use During Three Separate Waves of the COVID-19 Pandemic at a Large Academic Medical Center in Detroit, MI

**DOI:** 10.1093/ofid/ofab466.356

**Published:** 2021-12-04

**Authors:** Deepika Sivakumar, Shelbye R Herbin, Raymond Yost, Marco R Scipione

**Affiliations:** 1 Detroit Receiving Hospital, Detroit, Michigan; 2 Detroit Medical Center, Detroit, Michigan

## Abstract

**Background:**

Inpatient antibiotic use early on in the COVID-19 pandemic may have increased due to the inability to distinguish between bacterial and COVID-19 pneumonia. The purpose of this study was to determine the impact of COVID-19 on antimicrobial usage during three separate waves of the COVID-19 pandemic.

**Methods:**

We conducted a retrospective review of patients admitted to Detroit Medical Center between 3/10/19 to 4/24/21. Median days of therapy per 1000 adjusted patient days (DOT/1000 pt days) was evaluated for all administered antibiotics included in our pneumonia guidelines during 4 separate time periods: pre-COVID (3/3/19-4/27/19); 1st wave (3/8/20-5/2/20); 2nd wave (12/6/21-1/30/21); and 3rd wave (3/7/21-4/24/21). Antibiotics included in our pneumonia guidelines include: amoxicillin, azithromycin, aztreonam, ceftriaxone, cefepime, ciprofloxacin, doxycycline, linezolid, meropenem, moxifloxacin, piperacillin-tazobactam, tobramycin, and vancomycin. The percent change in antibiotic use between the separate time periods was also evaluated.

**Results:**

An increase in antibiotics was seen during the 1st wave compared to the pre-COVID period (2639 [IQR 2339-3439] DOT/1000 pt days vs. 2432 [IQR 2291-2499] DOT/1000 pt days, p=0.08). This corresponded to an increase of 8.5% during the 1st wave. This increase did not persist during the 2nd and 3rd waves of the pandemic, and the use decreased by 8% and 16%, respectively, compared to the pre-COVID period. There was an increased use of ceftriaxone (+6.5%, p=0.23), doxycycline (+46%, p=0.13), linezolid (+61%, p=0.014), cefepime (+50%, p=0.001), and meropenem (+29%, p=0.25) during the 1st wave compared to the pre-COVID period. Linezolid (+39%, p=0.013), cefepime (+47%, p=0.08) and tobramycin (+47%, p=0.05) use remained high during the 3rd wave compared to the pre-COVID period, but the use was lower when compared to the 1st and 2nd waves.

Figure 1. Antibiotic Use 01/2019 to 04/2019

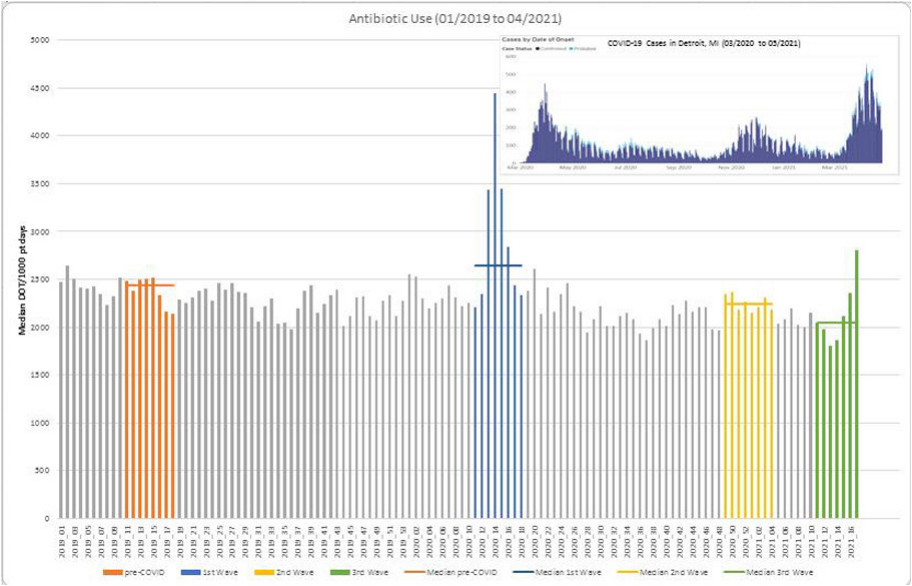

**Conclusion:**

Antibiotics used to treat bacterial pneumonia during the 1st wave of the pandemic increased and there was a shift to broader spectrum agents during that period. The increased use was not sustained during the 2nd and 3rd waves of the pandemic, possibly due to the increased awareness of the differences between patients who present with COVID-19 pneumonia and bacterial pneumonia.

**Disclosures:**

**All Authors**: No reported disclosures

